# High-Frequency Ultrasound Focusing Using Low-Cost PMMA and PDMS Acoustic Lenses

**DOI:** 10.3390/mi17040414

**Published:** 2026-03-28

**Authors:** Mohammadamir Ghasemishabankareh, Zeyuan Hui, Francesc Torres, Núria Barniol

**Affiliations:** Departament Enginyeria Electrònica, Escola d’Enginyeria, Universitat Autònoma de Barcelona, Bellaterra, 08193 Cerdanyola del Vallès, Spain; zeyuan.hui@autonoma.cat (Z.H.); francesc.torres@uab.cat (F.T.); nuria.barniol@uab.cat (N.B.)

**Keywords:** ultrasound, focusing, Fresnel lens, convex lens, MEMS, PMUT array

## Abstract

This study presents a high-frequency ultrasound lens system that uses simply fabricated and low-cost acoustic lenses made from PMMA and PDMS materials. These lenses are designed for higher-frequency operation around 20 MHz, providing suitability for demanding high-frequency ultrasonic applications. They were designed and fabricated specifically for integration with a PMUT array, ensuring proper compatibility with array-based high-frequency ultrasonic imaging. Both Fresnel and convex lens designs were evaluated through axial and lateral beam measurements, along with pulse–echo testing in the focal region. The results show that the PMMA and PDMS lenses can produce a well-defined focus and a stable echo response despite their simple and low-cost fabrication. This demonstrates the feasibility of low-cost materials for high-frequency ultrasonic focusing in PMUT array applications.

## 1. Introduction

In recent years, several diagnostic and therapeutic techniques have been developed in the medical field. These techniques are based on magnetic, microwave, optical, or ultrasonic principles. Some of these methods may have harmful effects on human tissues. For example, excessive use of X-rays in medical imaging can damage cells, and MRI can pose risks for patients with metallic implants. In addition, the purchase, maintenance, and repair of such equipment are associated with high costs. For these reasons, non-destructive approaches, particularly those based on ultrasound waves, are considered more suitable alternatives [1]. Ultrasound-based methods are widely used for diagnosis, imaging, and treatment because they are relatively low-cost and have fewer side effects [1,2]. Accordingly, advances in microelectromechanical systems (MEMS) have led to the development of micromachined ultrasonic transducers, including piezoelectric micromachined ultrasonic transducers (PMUTs) and capacitive micromachined ultrasonic transducers (CMUTs) [2,3].

PMUTs and capacitive micromachined ultrasonic transducers (CMUTs) are generally smaller and require less power than conventional bulk piezoelectric transducers [4]. These devices can be arranged in large arrays and integrated with electronic circuits through standard micromachining techniques, which makes them attractive for use in modern ultrasonic systems [5]. In recent studies, PMUT and CMUT devices have been operated at higher ultrasonic frequencies in order to improve imaging resolution [4,6]. However, higher operating frequencies lead to increased acoustic attenuation in water and biological tissues, which reduces both transmitted and received signal amplitude [4,6]. This effect is particularly pronounced in pulse–echo systems, where a single transducer is used for both transmission and reception.

Fresnel acoustic lenses focus ultrasound waves by means of constructive interference from concentric zones and have a flat, thin geometry, which makes them suitable for high-frequency applications [7,8]. Convex acoustic lenses can also provide effective focusing; however, their three-dimensional geometry generally results in a bulkier structure compared with Fresnel lenses [9]. Previous studies have shown that Fresnel lenses can increase acoustic pressure and enhance ultrasound imaging performance [10].

The combination of acoustic lenses with micromachined ultrasonic transducers has been studied by several groups. The results show that acoustic lenses reduce beam divergence and can partly compensate for the low output pressure of small-aperture transducers [11,12,13]. This can be achieved without modifying the transducer’s structure. For this reason, acoustic lenses are a practical and low-cost approach to addressing the limitations of PMUTs at high operating frequencies [11,12,13,14].

For high-frequency acoustic lenses, the choice of material and fabrication method is important. Polymethyl methacrylate (PMMA) and Polydimethylsiloxane (PDMS) are widely used for acoustic applications. Both materials have a speed of sound with enough different values with respect to the speed of sound in water to produce the focalization effect [15]. They have acceptable acoustic and mechanical properties and can be fabricated at a relatively low cost [15,16]. PMMA is a rigid material with good mechanical strength, and it is clearly suitable for using laser cutting or Computer Numerical Control (CNC) machining to fabricate the Fresnel lens structures. PDMS is suitable to be molded and has an acoustic impedance close to that of water, which helps reduce reflections at the lens interface [17]. Because of this, PDMS is often used for convex acoustic lenses operating at high frequencies [15,16].

The integration of acoustic lenses with PMUT arrays presents several challenges, including alignment accuracy, acoustic coupling, and impedance matching, which may affect the overall focusing performance. Therefore, in this work, the focus is placed on the design and characterization of acoustic lenses as standalone structures. Two acoustic lenses, a Fresnel lens and a convex lens, are fabricated and characterized, both designed to operate at 20 MHz and to focus the acoustic beam at a distance of 17 mm, making them suitable for various medical applications and compatible with PMUT arrays. The integration of these lenses with PMUT arrays will be addressed in future work, where a complete system will be developed and experimentally validated.

After the simulations and design stages, the lenses are fabricated and tested. Their focusing behavior is evaluated using two steps: firstly, detection of the position of the focal point in the z direction using a hydrophone, and secondly, characterization of the acoustic field using two-dimensional measurements (XZ plane). Pulse–echo measurements are then performed to study object detection using targets with two different diameters. Furthermore, the field analysis of the focal point under transmission-mode operation is used to evaluate the lens behavior at a focal length of 17 mm. These measurements allow a direct comparison between the Fresnel and convex lenses under the same conditions.

The following sections describe the lens design, fabrication process, experimental setup, and measurement results, followed by a discussion and conclusion.

## 2. Theory and Design of Acoustic Lens

The design of the lenses is examined in this section. Two lens configurations are considered: a Fresnel acoustic lens and a convex acoustic lens. Both lenses are designed to operate at a frequency of 20 MHz with a focal length of 17 mm in water, allowing their focusing behavior to be directly compared. The Fresnel lens offers the advantage of a planar structure, which simplifies integration with MEMS devices; however, its performance is limited by the decreasing ring width toward the outer zones and by fabrication–resolution constraints, which restrict the number of achievable rings. Conversely, the convex lens can overcome these limitations since its focusing capability is defined by the radius of curvature rather than discrete rings, but it requires a larger volume, which may be undesirable for compact systems. Due to these respective advantages and disadvantages, both lens types are considered in this work. PMMA (mechanically strong and suitable for machining) and PDMS (soft and easily moldable) are used for the design and fabrication of the Fresnel lens and the convex lens, respectively, which simplifies the fabrication process and reduces the overall fabrication cost.

### 2.1. Design of the Fresnel Lens

**Figure 1:**
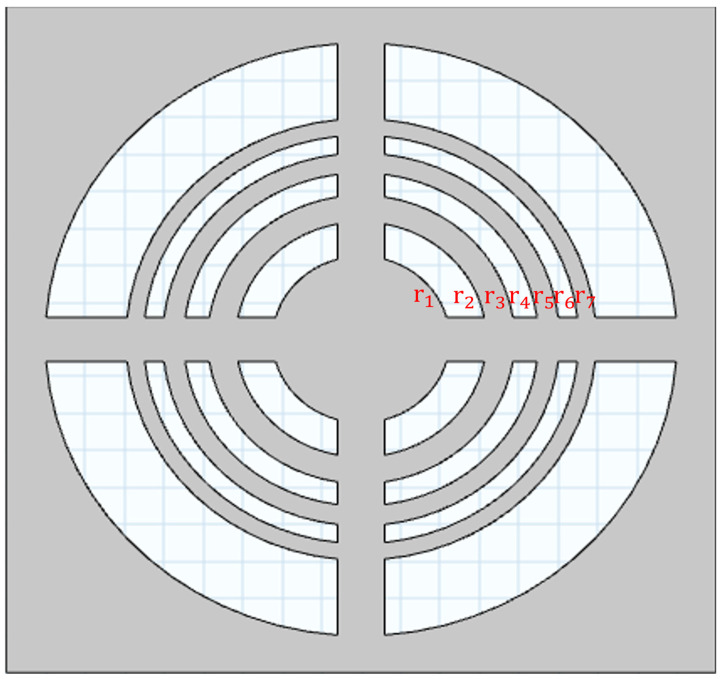
Schematic of Fresnel lens.

A Fresnel lens is a flat structure that can be used to focus ultrasonic waves. The lens is made of a few concentric circular zones. These zones are arranged in such a way that the acoustic waves passing through different parts of the lens reach the focal point with constructive interference. When the acoustic waves from its concentric zones arrive at the focal point with the same phase, their pressure amplitudes add coherently and amplify the focused field. In this case, focusing can be achieved using a Fresnel lens [7,16].

(1) $$r_{n}=\sqrt{n\lambda_{d}F+{\left(\frac{n\lambda_{d}}{2}\right)}^{2}},\;\;\;n=1,\ldots ,N$$

where $r_{n}$ is the radius of the zone; F is the focal distance; $\lambda_{d}$ is the wavelength in the surrounding medium, and n is the zone number. This equation allows the opaque zones to efficiently block the ultrasound waves. The required thickness $t_{h}$ of the Fresnel lens is selected so that the phase shift introduced in the opaque zones with respect to the transparent regions is an odd multiple of π. This condition is based on the difference between the wavelengths in the surrounding medium and in the lens material [16]. It can be expressed as

(2) $$t_{h}=\frac{q}{2}\frac{\lambda_{d}\lambda_{m}}{\left|\lambda_{d}-\lambda_{m}\right|}$$

where $\lambda_{d}\;$ is the acoustic wavelength in the surrounding medium and $\lambda_{m}$ the wavelength in the lens material. The parameter *q* = 1, 3, 5… controls the thickness assigned to the Fresnel regions in the lens design. Based on these design relations, the main geometric parameters of the Fresnel acoustic lens were calculated for fabrication. These parameters are summarized in Table 1, and the schematic of the Fresnel lens is illustrated in Figure 1. PMMA was selected for the Fresnel lens because its rigidity and machinability allow the formation of well-defined phase-stepped zones required for a thin and stable Fresnel plate structure.

### 2.2. Design of the Convex Lens

Acoustic waves exhibit behavior closely analogous to optical waves. When ultrasound propagates from one medium into another with a different speed of sound, its direction changes according to Snell’s law. It appears that the degree of refraction depends on the ratio of acoustic velocities in the two materials. Under the paraxial (small-angle) approximation, this refraction behavior leads to a quantitative relationship that defines the geometry of an acoustic lens, where the radius of curvature (*ROC*) is given by [4,17]

(3) $$ROC=F_{geo}*\left(\frac{C_{medium}}{C_{lens}}-1\right)$$

with $F_{\mathrm{geo}}$ denoting the geometric focal length of the lens, $C_{\mathrm{medium}}$ representing the speed of sound in the surrounding medium (e.g., water), and $C_{\mathrm{lens}}$ referring to the speed of sound within the lens material. Based on this curvature, the lens thickness (*TL*) representing the maximum depth the convex lens is defined by

(4) $$TL=ROC-\sqrt{{ROC}^{2}-{\left(\frac{D}{2}\right)}^{2}}$$

where *D* is the transducer diameter. The depth of view (*DOV*) describes the axial range of effective focusing and is given as follows, with λ denoting the acoustic wavelength in the medium. The value of 7.1 derives straight from the diffraction expression given that for a circular aperture the on-axis intensity follows a sinc dependence, and the −6 dB points are located at $z_{\pm }=\pm 3.55\;\lambda (F/D{)}^{2}$. The constant therefore can be obtained to describe the complete separation of these two symmetric −6 dB positions, written as 2×3.55.

(5) $$DOV=7.1*\lambda *{\left(\frac{F_{geo}}{D}\right)}^{2}$$

**Figure 2:**
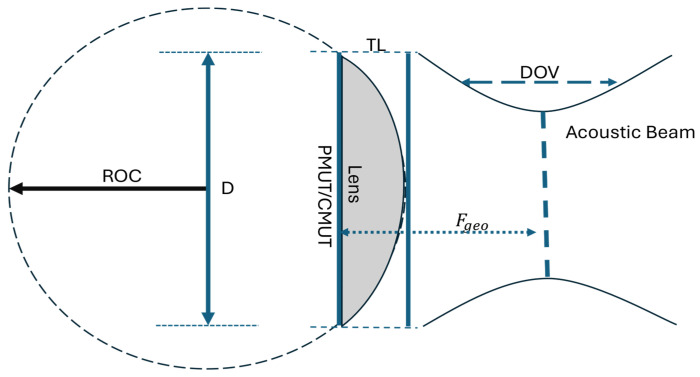
Schematic of convex lens.

PDMS was paired with a convex geometry since its moldability and acoustic impedance close to water make it well suited for forming smooth refractive profiles with low interface reflection, and due to its lower sound speed relative to water, the PDMS structure naturally behaves as a converging (convex) acoustic element; the corresponding geometric and acoustic parameters are summarized in Table 2 and Figure 2.

## 3. Lens Simulation and Fabrication

In this section, based on the parameters mentioned in Section 2, the two lenses are evaluated using the finite element method. After validating the simulation results, the Fresnel lens and the convex lens are fabricated using a simple procedure.

### 3.1. Fresnel and Convex Lens Simulaion

**Figure 3:**
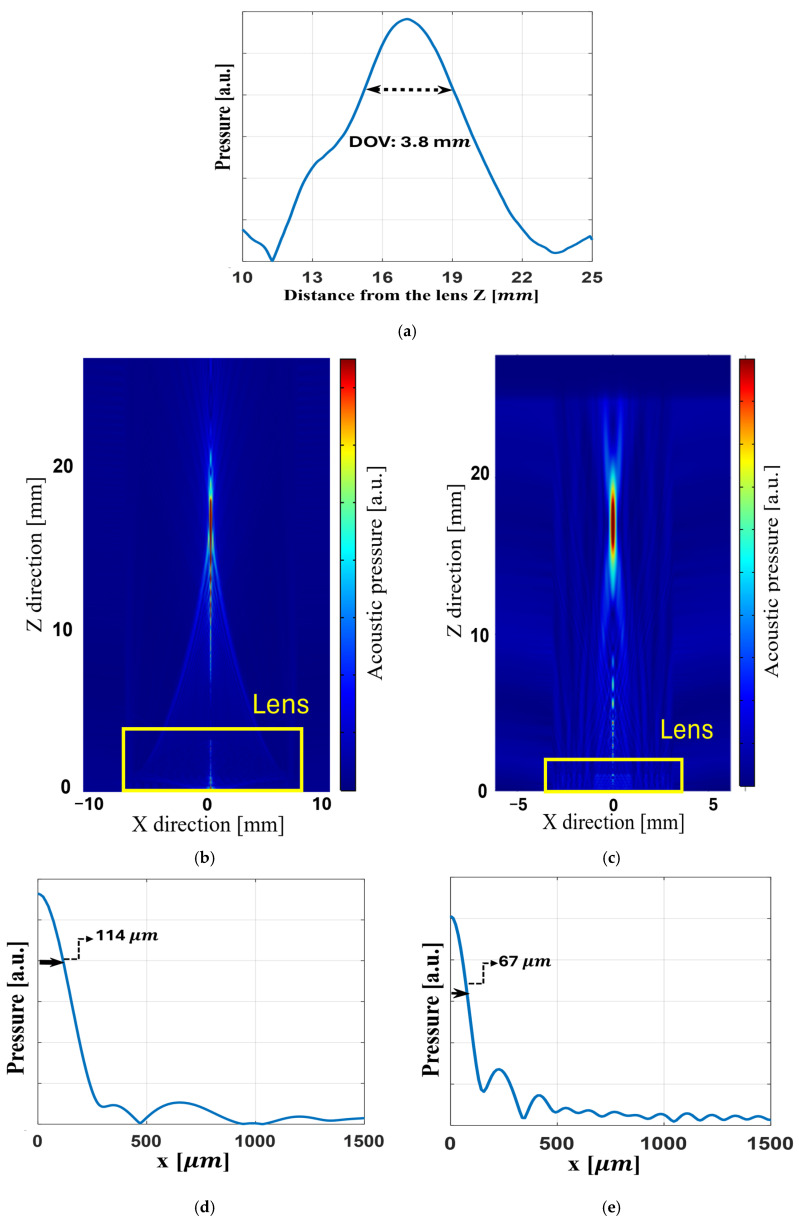
COMSOL FEM study: (**a**) axial pressure profiles in Z direction of the Fresnel focusing field; (**b**) Fresnel lens focusing field; (**c**) convex focusing field; (**d**) pressure profiles in x direction for Fresnel lens focusing field; (**e**) pressure profiles in x direction for convex lens focusing field.

The performance of the two lenses, namely the Fresnel lens and the convex lens, was evaluated using the finite element method (FEM) implemented in COMSOL Multiphysics 6.3. The COMSOL Multiphysics simulations are performed over a 2D model, and the plots are extracted from this 2D simulation. Furthermore, Figure 3 allows us to evaluate the performance of the Fresnel lens and convex lens.

Figure 3a indicates that at a 17 mm focal depth of the Fresnel lens, the DOV of the Fresnel lens is about 4 mm. Figure 3b,c clearly show the 2D acoustic field of both lenses. Furthermore, the half resolution of the Fresnel lens and convex lens at the focal point is 134 µm and 228 µm, respectively; these values are shown in Figure 3d,e. These resolutions and DOV in Figure 3a were determined based on the −3 dB amplitude threshold, corresponding to an amplitude ratio of 0.707 with respect to the maximum pressure. The simulation of both lenses operating at 20 MHz in water confirms that the acoustic waves converge precisely at this intended focal distance. The results of lenses show their reliable and effective performance prior to fabrication.

### 3.2. Fresnel Lens Fabrication

Validating the lens behavior using the FEM approach demonstrates that the lenses are ready for fabrication. Both lenses were produced using simple and low-cost techniques. PMMA was selected for the Fresnel lens. This material is inexpensive and provides sufficient mechanical strength for fabrication using CNC milling or laser-cutting systems. PDMS is a good candidate for the convex lens because it can be rapidly molded through straightforward casting and curing processes. These material selections enable both lenses to be manufactured efficiently and with minimal complexity. The following sections describe the fabrication procedures in detail.

**Figure 4:**
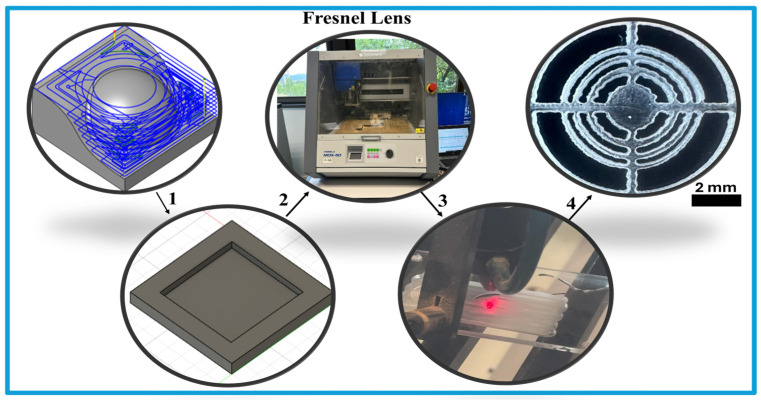
Fabrication process of the Fresnel lens: (1) 3D pocket machining, (2) pool-structure milling, (3) laser cutting, and (4) final lens (scale 2 mm).

The Fresnel lens is designed for an operating frequency of 20 MHz and a focal distance of 17 mm. The fabrication process is presented as follows:

Figure 4 illustrates the main steps involved in fabricating the Fresnel lens. The process begins with 3D pocket milling to create a pool on the PMMA substrate. This step prepares the base geometry and the required thickness for the Fresnel zones. After milling using a CNC machine, the Fresnel pattern is directly transferred onto the PMMA surface using a laser-cutting machine. This procedure results in the final lens with the intended concentric features. The small edge roughness observed in Figure 4 can be considered a minor random perturbation that slightly redistributes energy into weak side lobes, which is produced by diffraction. However, because it does not introduce systematic phase errors, the main constructive interference remains intact. As confirmed in Section 5 and Section 5.2.1, the focusing performance of the lens is preserved.

### 3.3. Convex Lens Fabrication

**Figure 5:**
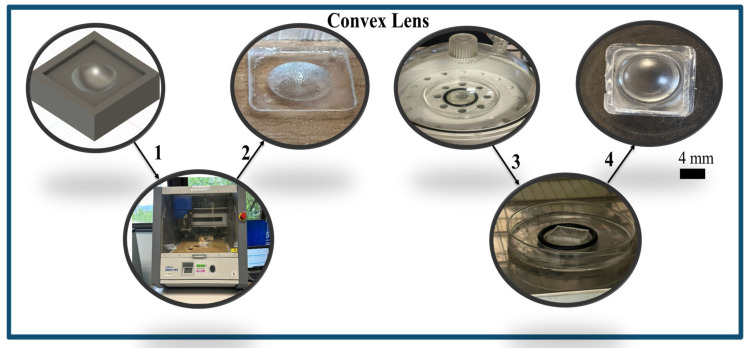
Convex lens fabrication process: (1) mold milling, (2) Vaseline coating, (3) PDMS degassing and curing on PMMA sheet, (4) lens release (scale 4 mm).

A convex acoustic lens designed to operate in the megahertz frequency range is fabricated using a simple and inexpensive method based on PDMS used as the base material. The fabrication process is described as follows:

The fabrication process takes several steps, based on Figure 5. To begin, the concave mold shape is milled on the PMMA. A very thin film of Vaseline is then applied to the mold to prevent the PDMS from sticking during curing. The PDMS is subsequently degassed in a vacuum chamber on the PMMA as a base with a thickness of 4 mm; the mold is inversely mounted to the PDMS, and the sample is cured at 100 °C in an oven for 5 h. The mold is subsequently removed after curing to achieve the convex PDMS lens.

## 4. Characterizing the Convex and Fresnel Lenses

In this section, the performance of the acoustic lenses is examined. Two types of experiments are designed to evaluate the behavior of the lenses. In the first experiment, the lens performance is analyzed using a commercial hydrophone while generating ultrasound waves through a commercial transducer to identify the focal point position along the Z axis (normal to the transducer surface). After that, the ultrasound pressure at the focal point is measured in the XZ plane. In the second experiment, the pulse–echo technique is used to detect an obstacle placed at the focal point. This method allows the assessment of the lens performance as an ultrasound focusing element and demonstrates its accuracy and efficiency.

### 4.1. Experimental Setup and Measurement of Convex and Fresnel Lenses

**Figure 6:**
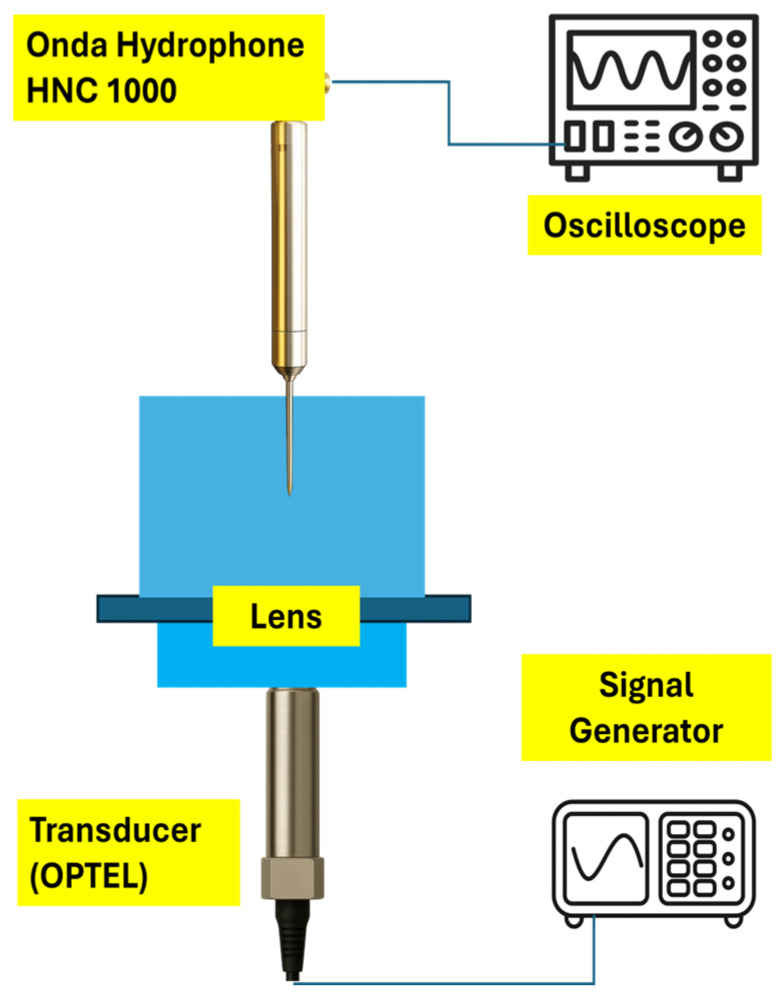
Acoustic measurement experimental setup for finding the focal point and performing two-dimensional measurement.

In this measurement two lenses were evaluated using the same experimental setup. A sinusoidal signal was generated by a Keysight 81150A signal generator (Santa Rosa, CA, USA) with a peak-to-peak voltage of 20 V at a frequency of 20 MHz. This signal was applied to a commercial ultrasound transducer from OPTEL (Wrocław, Poland) [18], with a nominal center frequency of 20 MHz and diameter of 6 mm. The transducer converted the electrical input into an ultrasound signal at the same frequency. As we can see in Figure 6, a convex lens and a Fresnel lens were immersed in front of the transducer. Under these conditions the lenses were capable of focusing the ultrasound signal at a distance of 17 mm. An Onda HNC 1000 hydrophone (Sunnyvale, CA, USA) [19] was employed to measure the ultrasound signal in order to search for focal points and perform two-dimensional measurement along the XZ plane. The acquired signals were finally recorded and analyzed using a Keysight InfiniiVision DSOX3054A oscilloscope (Santa Rosa, CA, USA).

### 4.2. Pulse–Echo Measurement with the Fresnel Lens Operating as Sensors

**Figure 7:**
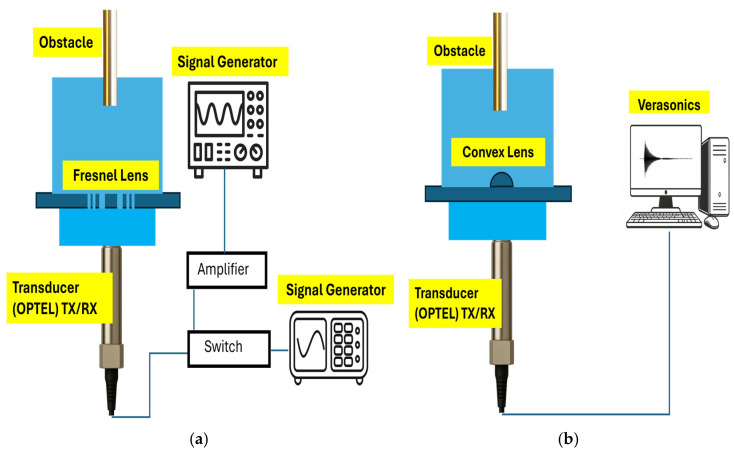
Pulse–echo measurement setup; (**a**) Fresnel lens; (**b**) convex lens.

The OPTEL transducer was used both as a transmitter and as a receiver to generate ultrasonic waves and detect the echo reflected from an obstacle positioned in front of the lens at a different distance, which is sweeping in the Z direction. In this configuration, the Fresnel lens is placed 9 mm in front of the OPTEL transducer and connected to an RF switch, which enables alternating operation of the transducer as a source and as a detector. However, because this transducer operates primarily as a source, the echo signal it receives is very weak, and an amplifier is required to improve the detection of the reflected ultrasound. As the lens is made of PMMA, this acoustic wave experiences reflections at both the front and rear PMMA interfaces, which results in different time-of-flight (ToF) parts in the received signal. When the transducer is located too close to the lens, the ToF of these interfaces becomes very short and overlaps with the ToF of the obstacle, inhibiting the system from detecting the response of the obstacle effectively. Increasing the distance between the transducer and lens results in longer time-of-flight (ToF) of the PMMA-related components but also increases propagation distance within PMMA, thus leading to increased acoustic attenuation and decreased signal amplitude. A spacing of 9 mm is thus optimal because it balances sufficient ToF separation with enough signal strength to enable reliable obstacle detection. Moreover, due to the fact that the echo signal received is low and that attenuation is introduced in the case of the convex lens due to the presence of the PMMA, the pulse–echo method requires a higher input signal compared to that used in the case of the Fresnel lens. For the case of the Fresnel lens, the maximum possible signal that can be supplied by the signal generator, which is 20 Vpp, is enough. For the case of the convex lens, however, a clear echo requires a higher input signal, which is 30 Vpp, compared to the output of the signal generator. The setup of the echo pulse measurement related to the Fresnel lens setup is presented in Figure 7a.

### 4.3. Pulse–Echo Measurement with the Convex Lens Operating as Sensors

Since the OPTEL is used simultaneously as both a transmitter and a receiver, the received signal in the OPTEL is partially attenuated. To compensate for this issue, a commercial Verasonics system is employed for signal transmission and reception via the OPTEL, such that the signal received by the OPTEL can be easily detected by the Verasonics system. The Verasonics system includes a switch and amplifier, which can help in detecting a signal. In this measurement, a peak-to-peak voltage of 30 V and a frequency of 20 MHz are used for transmitting and receiving the signal through OPTEL. A convex lens is mounted on a PMMA plate with a thickness of 4 mm and positioned at 9 mm from the OPTEL, and a metal circular obstacle with a diameter of 6 mm is used for sweeping at different distances in the Z direction. The setup of this measurement is shown in Figure 7b.

### 4.4. Pulse–Echo Detection for Small Objects via Convex Lens

In this measurement, the detection of an obstacle located within the focal region via a convex lens is investigated. The obstacle considered is copper covered by plastic with a diameter of 1.9 mm, positioned 17 mm away from the convex lens and moving laterally across the width of the focal zone. The echo signal reflected from the copper covered by plastic is received and detected by OPTEL. In this experiment, the OPTEL is employed for both transmission and reception of the acoustic signal, while the excitation signal is generated by the Verasonics system. The Verasonics system produces a 30 V peak-to-peak signal at a frequency of 20 MHz for this measurement. This measurement employed the same experimental setup described in Section 4.2.

### 4.5. Effect of a Small Object at the Convex Lens Focal Point (Transmission Mode)

**Figure 8:**
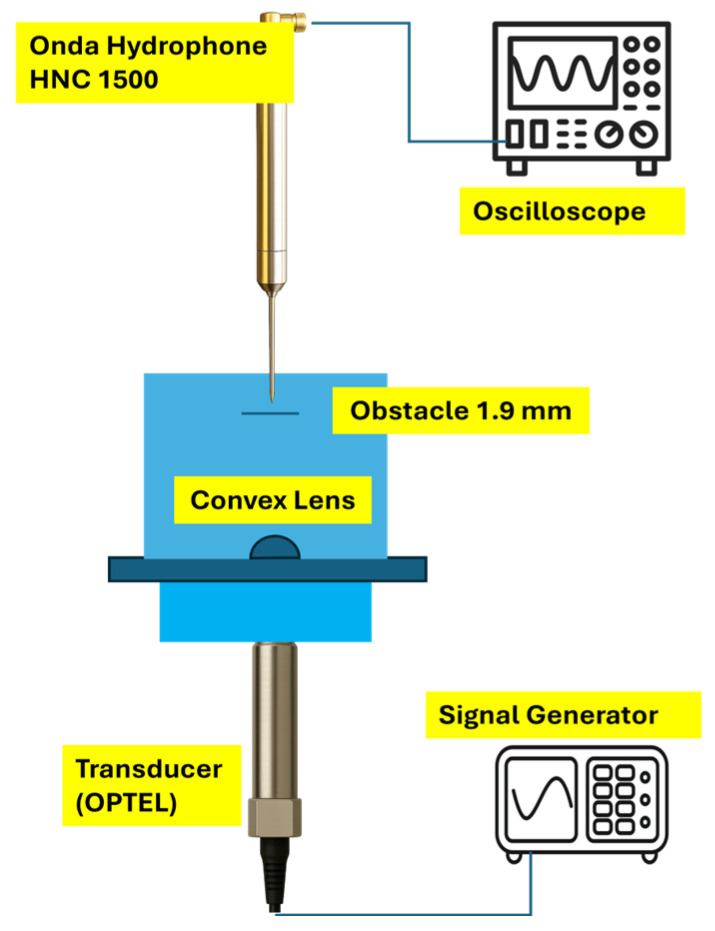
Measurement setup to characterize the object profile pressure.

In this experiment, a cable-shaped obstacle with a diameter of 1.9 mm was placed at the focal point of the lens. The lens was mounted 9 mm from the transmitting element OPTEL as transducer. The transducer was driven by Verasonics, which generated a 20 MHz sine wave with an amplitude of 30 V peak-to-peak. All measurements were performed in transmit mode, and the pulse–echo configuration was not used. An Onda HNC-1500 hydrophone (Sunnyvale, CA, USA) was employed to detect the acoustic field transmitted through the lens and the obstacle. The hydrophone was positioned 18 mm from the obstacle and moved horizontally and transversely across the focal region to record the spatial distribution of the ultrasonic pressure field. The acquired signals were monitored and recorded using a Keysight InfiniiVision DSOX3054A oscilloscope. The general experimental setup is shown in Figure 8.

## 5. Outcomes

The results of this work are divided into two main parts. The first part focuses on how each lens behaves when used for acoustic focusing at high frequency. This part describes the ability of the structure to form a clear focal region and how the field evolves around that area. The second part looks at the role of each lens as a sensing element. In this case the analysis relies on pulse–echo measurements that reflect the strength and clarity of the detected signals. These two parts together offer a broader view of the performance of the PDMS convex lens and the PMMA Fresnel lens in practical use.

### 5.1. Characterizing the Behavior of the Convex and Fresnel Lenses

**Figure 9:**
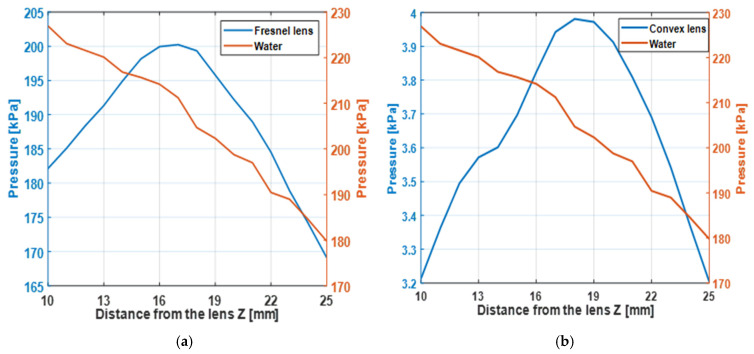
Acoustic pressure along the Z-direction for determining the focal point: (**a**) Fresnel lens; (**b**) convex lens.

**Figure 10:**
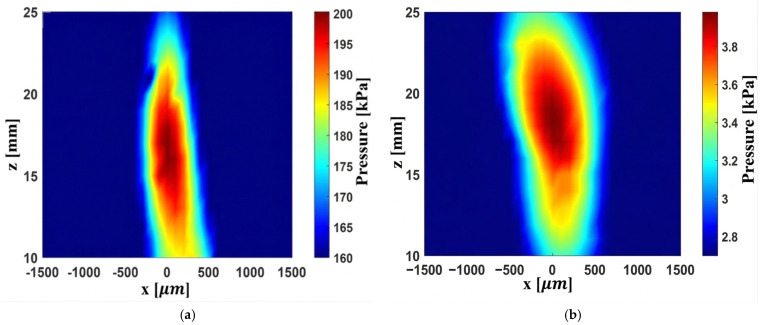
2D map of acoustic fields; (**a**) Fresnel lens; (**b**) convex lens.

**Figure 11:**
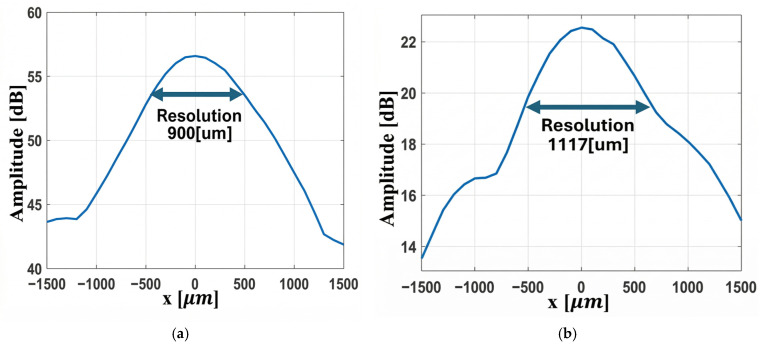
Lateral zone width at focal point; (**a**) Fresnel lens; (**b**) convex lens.

The Fresnel and convex lenses were positioned 9 mm from the transducer, and the acoustic field at 20 MHz was measured using a hydrophone. The axial focal position at 17 mm is shown in Figure 9. The two-dimensional focal spot maps of both lenses are presented in Figure 10, and the lateral beam width is illustrated in Figure 11.

In Figure 9a, the Fresnel lens exhibits a higher maximum pressure of 200 kPa, whereas Figure 9b shows that the convex lens generates a significantly lower pressure of approximately 3.99 kPa.

Figure 10a shows that at 20 MHz, the Fresnel lens focuses sound at the 17 mm focal point with a higher acoustic pressure, whereas Figure 10b demonstrates that the convex lens also focuses at the same distance but produces a lower peak pressure. To clarify the origin of this difference, the attenuation and transmission properties of the materials must be analyzed.

First, the attenuation characteristics were evaluated. Acoustic pressure in water was measured as a reference using an Onda hydrophone 1000 at a fixed position relative to the transducer. Then, PMMA and PDMS samples with a thickness of 3 mm were placed 9 mm from the transducer, and the transmitted pressure was recorded at the same hydrophone location. The pressure reduction relative to the water reference was used to calculate insertion loss. The results show that PMMA exhibits a relatively high attenuation of 5.19 dB/mm, while PDMS has a lower attenuation coefficient of 2.25 dB/mm. However, in the convex lens configuration, which consists of 2.7 mm PDMS and 4 mm PMMA, the total attenuation accumulates to approximately 37 dB due to the increased propagation path through both materials.

Next, the transmission behavior was analyzed in relation to acoustic impedance mismatches. As reported in Table 2, the acoustic impedances of PMMA, PDMS, and water are 3.3 MRayl, 1.0 MRayl, and 1.5 MRayl, respectively. Because PDMS has an acoustic impedance closer to that of water, the water–PDMS–water configuration yields transmission coefficients of approximately 0.8 and 1.2, resulting in an overall transmission of about 0.96, indicating efficient energy transfer. In contrast, the water–PMMA–water configuration produces transmission coefficients of approximately 1.37 and 0.62, leading to a lower overall value of about 0.86, reflecting stronger impedance mismatch effects. For the combined water–PDMS–PMMA–water structure, the total transmission decreases to approximately 0.75.

Although PDMS improves impedance matching with water, the convex lens geometry introduces a longer propagation path through both PDMS and PMMA layers. This results in increased cumulative attenuation, which has a big impact on the transmission of the ultrasound wave.

As illustrated in Figure 11a,b, the lateral focal width of the Fresnel lens is smaller than that of the convex lens, i.e., 900 µm vs. 1117 µm at the −3 dB level. The 2D FEM simulations yield much smaller lateral widths for both lenses, i.e., approximately 228 µm for the Fresnel and 134 µm for the convex lens. This is understandable, as the simulations are based on an ideal detector, without instrument effects, directivity, and reverberations. Conversely, the measurements include all these effects. The Onda HNC-1000 hydrophone with nominal tip/aperture of 1 mm produces spatial averaging effects, effectively convolving the measurement response with the spatial response of the hydrophone, with a final lateral width close to the nominal 1 mm size of the hydrophone. Using a smaller hydrophone needle tip will reduce spatial averaging effects and give an accurate measurement of the lateral width. The 2D FEM correctly predicts the instrument-independent fields, accurately locating the focal position at 17 mm, and the ideal resolution, while the measurements give an instrument-dependent, realistic response. The focusing ability of both lenses is quite strong, and the Fresnel lens produces a brighter, more compact focal spot.

### 5.2. Lens Sensing Behavior via Pulse–Echo

In this section, the results of the sensing performance are examined using the pulse–echo technique. Both the Fresnel lens and the convex lens are evaluated with this method; the analysis begins with the Fresnel lens and is then followed by a full assessment of the convex lens.

#### 5.2.1. Study of Fresnel Lens Behavior as a Sensor

**Figure 12:**
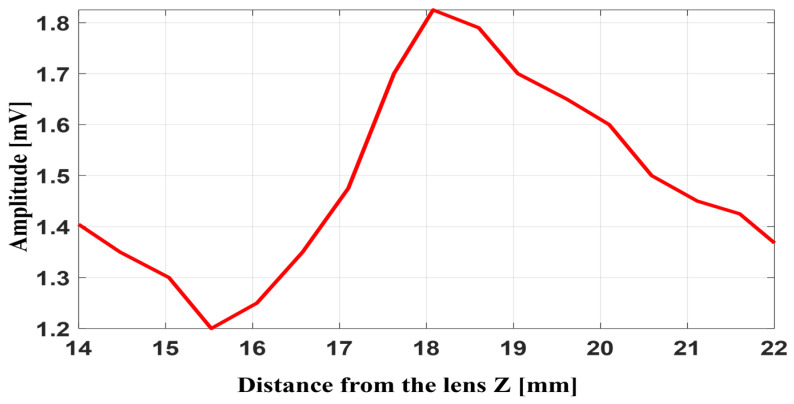
Pulse–echo detection of an object at varying distances from the lens.

In this experiment, the pulse–echo method is used to evaluate the performance of the Fresnel lens at high ultrasound frequencies. The 20 MHz transducer sends the pulses and receives the returned signals. The lens is placed about 9 mm in front of the transducer. A circular target with a diameter of 6 mm is positioned over the central axis of the lens and is moved step by step along the Z axis. As the distance changes, the echo intensity also changes. The strongest echo appears at about 17 mm from the lens, and this distance is considered the focal position. The amplitude of echo detection of objects at different distances from the lens and system echo responses is shown in Figure 12.

**Figure 13:**
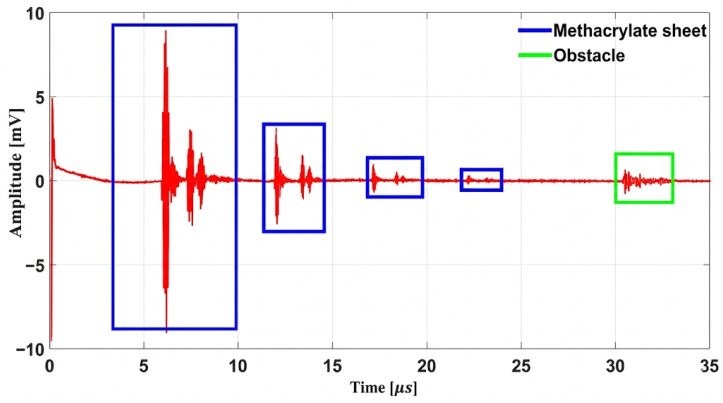
Pulse–echo detection of the Fresnel lens system.

Figure 12 shows the pulse–echo performance of the Fresnel lens. During this test, a 6 mm target is translated along the *Z* axis, and the echo amplitude is recorded at each position. The maximum echo occurs at approximately 17 mm with value of 1.82 mV, matching the lens’ focal distance and indicating optimal object detection. The pulse–echo detection of the whole system is shown in Figure 13.

Figure 13 presents the pulse–echo response of the overall system. In this plot, the reflections originating from the PMMA and the lens surfaces are observed at approximately 5.88 µs, corresponding to a propagation distance of about 9 mm from the transducer. The four blue boxes highlight the surface echoes produced by the PMMA surfaces and the Fresnel lens, respectively. The green box delineates the echo produced by the object at 30 µs, corresponding to a distance of 44 mm from the transducer. These results demonstrate that the system can separate echoes originating from different distances and provide reliable sensing performance.

#### 5.2.2. Study of Convex Lens Behavior as a Sensor

**Figure 14:**
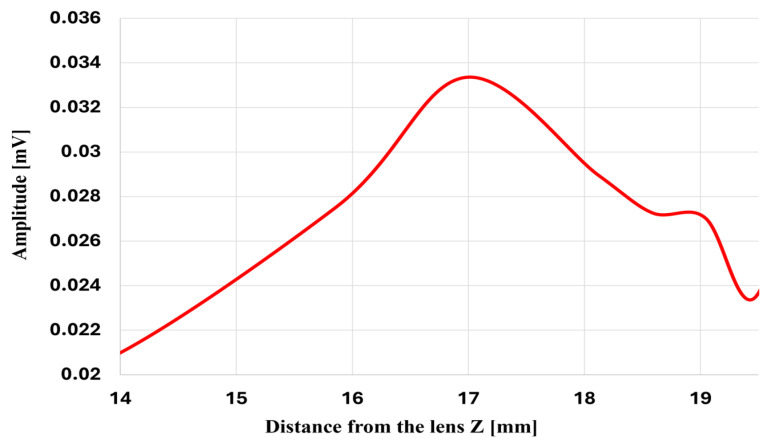
Pulse–echo detection with a convex lens as a function of the axial distance between the lens and the object.

**Figure 15:**
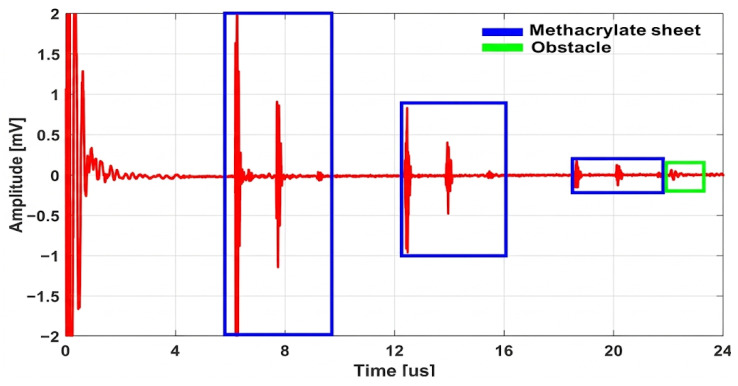
Time-domain response in pulse–echo detection with a convex lens.

In this experiment, the pulse–echo technique is applied using a Verasonics system connected to a transducer. The PMMA as base and lens is positioned 9 mm from the transducer, as in the previous setup, and an obstacle with a circular surface and a diameter of 6 mm is placed in front of the lens. The amplitude of the reflected signal from this obstacle is examined at various distances from the lens. The results obtained from this experiment are presented in Figure 14 and Figure 15.

Figure 14 shows the pulse–echo response of the convex lens. A 6 mm target was scanned along the *Z* axis, and the echo amplitude was measured at each position. The maximum echo amplitude of about 0.033 mV occurred near 17 mm, matching the lens’s focal length and indicating the point of optimal detection. Furthermore, the time-domain response of pulse–echo detection of the system is shown in Figure 15.

This figure shows the complete pulse–echo response of the system. These reflections are at approximately 6 µs from the 4 mm thick PMMA base and the convex lens, corresponding to their physical positions relative to the transducer. The echo from the obstacle can be detected at around 22 µs, which is found at a distance of 32 mm from the transducer. These results clearly demonstrate the presence of echoes at distinct time intervals.

#### 5.2.3. Field Analysis of the Convex Lens Focus Using a 1.9 mm Obstacle

**Figure 16:**
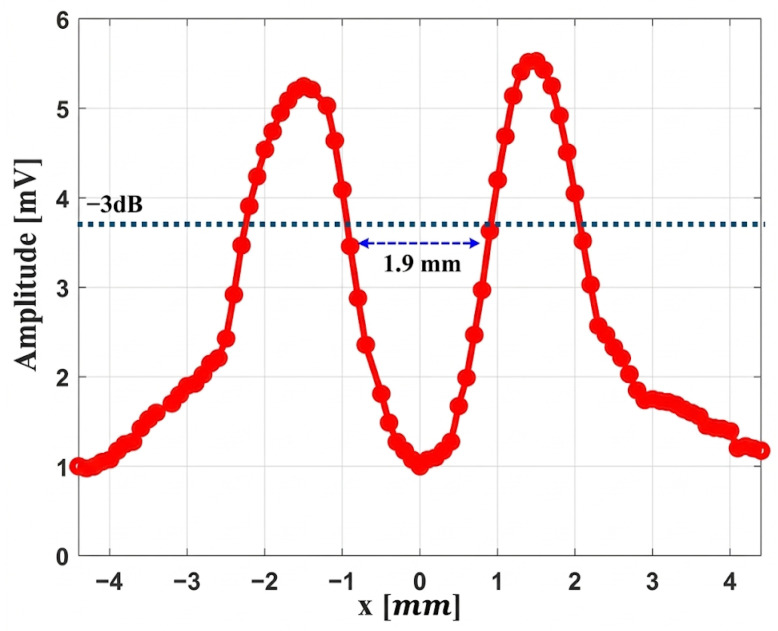
Acoustic wave distribution via object using the convex lens system.

In this experiment a cable with a diameter of 1.9 mm is placed 17 mm from the lens, which corresponds to the focal point of the lens. An Onda HNC-1500 hydrophone is used for this measurement and is positioned 18 mm from the object to detect the shadow effect of the acoustic beam behind the obstacle. After applying a 20 MHz ultrasound signal with an amplitude of 30 V peak-to-peak, the behavior of the acoustic field in the focal region is examined with the hydrophone. In this experiment, the obstacle remains fixed while the hydrophone moves laterally across the acoustic field (*x*-axis) to record variations in the signal across the focal width. The results of this scan are presented in Figure 16.

This figure shows the diffraction pattern created when the acoustic field encounters a 1.9 mm obstacle at the focal point based on the −3 dB criterion. The two peaks arise from the bending of the waves around the edges of the object, while the central depression is due to the acoustic shadow just behind it. This pattern is the typical result of wave diffraction around a small target.

#### 5.2.4. Focal Acoustic Field Analysis with a Convex Lens Sensor

**Figure 17:**
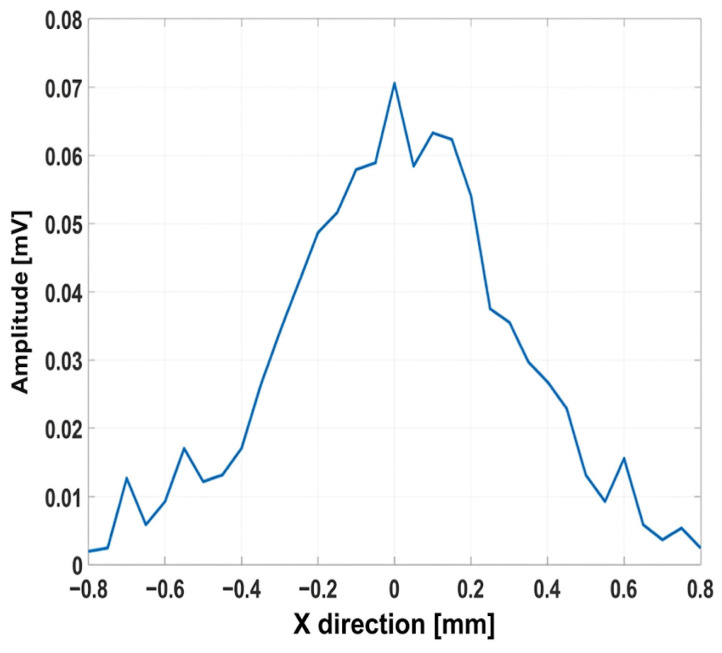
The amplitudes of the reflected echo at the focal point.

In this experiment, the convex lens is placed 9 mm in front of the transducer, which is used to both sense and receive the ultrasound waves. A small 1.9 mm diameter copper piece covered by plastic cable is positioned at the focal point of the lens and then moved step by step along the *X* axis (the lateral direction). At each position, the reflected ultrasound signal is recorded so the lateral pattern of the received waves can be examined. The amplitudes of the reflected echoes at the focal point for the 1.9 mm copper piece are presented in Figure 17.

**Figure 18:**
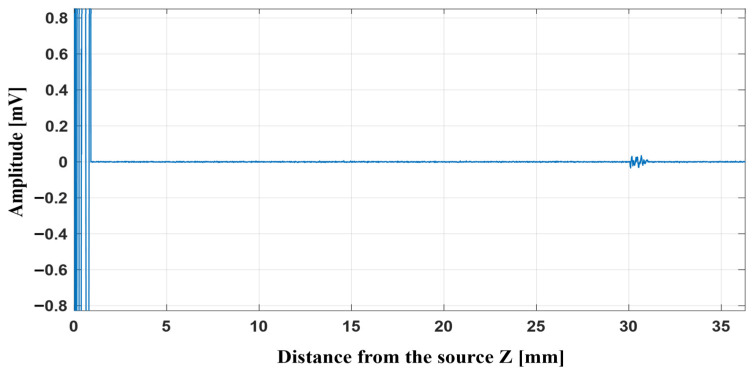
Reflected echo of copper covered by plastic at focal point with narrowband FIR band-pass filter.

Figure 18 shows the echo from the object in measurement. This obstacle is placed 30 mm from the source. The clear peak in the signal indicates that the object is well detected at this position. Operating at 20 MHz allows the system to create a sharp focus, so the reflected echo appears strong and easy to separate from other signals. As shown in Figure 18, a narrowband FIR band-pass filter was used to clean the signal. This type of filter removes unwanted noise and keeps only the main part of the echo, producing a clearer waveform in the graph.

As shown in Figure 17, the copper covered with plastic produces an echo of about 0.07 mV. The corresponding reflection echoes of this object are shown in Figure 18.

## 6. Conclusions

The results of this study demonstrate that both the PMMA Fresnel lens and the PDMS convex lens can achieve effective ultrasonic focusing at a high operating frequency of 20 MHz, despite being fabricated from very low-cost materials using extremely simple manufacturing methods. A similar focusing approach based on a PMUT array and a Fresnel-zone-plate structure was reported by Shimoyama et al. [20]; however, the lenses proposed in this work enable ultrasonic focusing at 20 MHz through simpler and more economical fabrication techniques. One of the most significant findings of this study is that high-frequency ultrasonic focusing does not necessarily require expensive materials or advanced microfabrication processes. Instead, readily available PMMA and PDMS, combined with straightforward methods such as laser cutting, CNC milling, and simple curing, are sufficient to achieve stable and high-quality acoustic performance.

The comparative characterization clearly shows that the PMMA Fresnel lens provides a stronger and more concentrated focus, producing higher acoustic pressure at the focal point. This can be attributed to both the Fresnel phase-matching geometry and the favorable acoustic behavior of PMMA, which has low attenuation and good mechanical rigidity, despite being one of the cheapest materials available. The lens produced a narrow lateral focal zone, confirming that even a very low-cost, easily machined PMMA lens can deliver high-precision focusing at 20 MHz.

In contrast, the PDMS convex lens, fabricated through a simple molding and curing process, also achieved the designed 17 mm focal distance. Although the peak pressure was lower, the PDMS lens demonstrated excellent impedance matching to water, which reduces reflection losses, and it showed strong capability to detect different object sizes—even very small ones. These results reinforce that PDMS is a high-performing yet inexpensive material for high-frequency acoustic applications, especially when ease of fabrication is a priority.

The pulse–echo experiments also verified that both inexpensive lenses can suit effective sensing components. The Fresnel lens was able to emit clear echoes at the focal region, and confirmed that simple and inexpensive materials can work very well for high-precision object detection. The diffraction patterns seen for the small targets corresponded to classical acoustic behavior in high-frequency observations corroborating the lens designs.

This work demonstrates that high-frequency ultrasonic lenses can be manufactured at low cost, using simple fabrication steps and widely available materials, without compromising focusing performance. PMMA and PDMS are practical, cheap, and acoustically suitable for the operating conditions of focusing and sensing even at 20 MHz. The results make it possible to achieve low-cost, high-performance ultrasound systems including MEMS-based transducers as well as small imaging devices.

## Figures and Tables

**Table 1:** Summary of parameters.

Parameters	Values	Parameters	Values
Operating Frequency	20 MHz	Density (PMMA)	1200 ${kg/m}^{3}$
Focal distance	17 mm	Young’s modulus (PMMA)	2855 MPa
Lens material	PMMA	Poisson’s ratio (PMMA)	0.4
Lens diameter	6 mm	$r_{1}$	1.1 mm
Number of radii	7	$r_{2}$	1.5 mm
$t_{h}$ (Thickness)	1 mm	$r_{3}$	1.9 mm
Speed of sound (PMMA)	2750 m/s	$r_{4}$	2.2 mm
Speed of sound (Water)	1500 m/s	$r_{5}$	2.5 mm
$\lambda_{0}$ (Water)	75 µm	$r_{6}$	2.7 mm
$\lambda_{m}\;$(PMMA)	140 µm	$r_{7}$	3 mm

**Table 2:** Summary of parameters for convex lens.

Parameters	Values	Parameters	Values
Operating Frequency	20 MHz	$\lambda_{0}$ (Water)	75 µm
Focal distance	17 mm	$\lambda_{m}$ (PDMS)	47.5 µm
Lens material	PDMS	Density (PDMS)	970 ${kg/m}^{3}$
ROC	10 mm	Young’s modulus (PDMS)	750 kPa
TL	2.7 mm	Poisson’s ratio (PDMS)	0.49
D	5 mm	Thickness as a Base (PMMA)	4 mm
DOV	6 mm	Acoustic impedance (PMMA)	3.3 MRayl
Speed of sound water	1500 m/s	Acoustic impedance (PDMS)	1.0 MRayl
Speed of sound PDMS	1050 m/s	Acoustic impedance (Water)	1.5 MRayl

## Data Availability

The original contributions presented in this study are included in the article. Further inquiries can be directed to the corresponding author.
